# Efficacy and safety of wet cupping in the treatment of neurodermatitis: a systematic review and meta-analysis

**DOI:** 10.3389/fmed.2024.1478073

**Published:** 2024-12-19

**Authors:** Yan Zhai, You-yi Hui, Ze-fei Jiang, Lin Ding, Jie Cheng, Tang Xing, Han Zhai, Hong Zhang

**Affiliations:** ^1^School of Acupuncture-Moxibustion and Tuina, Chengdu University of Traditional Chinese Medicine, Chengdu, China; ^2^Department of Gastroenterology and Hepatopathy, Xi’an Daxing Hospital, Xi’an, China; ^3^Sixth Department of Obstetrics (Foetal Protection Centre), Northwest Women’s and Children’s Hospital, Xi’an, China

**Keywords:** wet cupping, neurodermatitis, lichen simplex chronicus, efficacy, meta-analysis, systematic review

## Abstract

**Background:**

Neurodermatitis is a chronic skin condition characterized by intense itching and skin thickening due to neurological dysfunction. Its persistent nature poses a challenge to effective treatment, significantly impacting patients’ quality of life. Wet cupping therapy is increasingly being used in clinics to manage neurodermatitis, so it is imperative to assess the evidence regarding its effectiveness and safety.

**Objective:**

This review aimed to evaluate the efficacy and safety of wet cupping therapy in patients with neurodermatitis.

**Methods and analysis:**

Randomized controlled trials (RCTs) investigating wet cupping for neurodermatitis were identified through searches of eight electronic databases and three clinical trial registration platforms from inception to March 2024, using predefined search terms. Included studies underwent quality appraisal using the Cochrane Collaboration’s Risk of Bias Assessment tool. The quality of evidence was assessed independently by two reviewers using the Grading of Recommendations Assessment, Development, and Evaluation System (GRADE). Meta-analysis and publication bias assessment were conducted using ReviewManager 5.4 and STATA 17.0 software, respectively.

**Results:**

This review encompassed 19 studies, comprising 6 types of comparisons and involving 1,505 participants. The findings revealed no significant difference in the total effective rate between wet cupping alone and high-potency steroids (*n* = 269, RR = 1.13, 95% CI [0.90, 1.41], *p* = 0.29, I^2^ = 83%). However, wet cupping combined with medication or moxibustion exhibited superior efficacy compared to medication alone (*n* = 272, RR = 1.28, 95% CI [1.16, 1.41], *p* < 0.00001, I^2^ = 43%) and (*n* = 534, RR = 1.22, 95% CI [1.14, 1.30], *p* < 0.00001, I^2^ = 0%). Wet cupping groups demonstrated lower recurrence rates (*n* = 266, RR = 0.31, 95% CI [0.16, 0.60], *p* = 0.0005, I^2^ = 0%) and a reduced incidence of adverse events (*n* = 673, RR = 0.44, 95% CI [0.21, 0.90], *p* = 0.02, I^2^ = 36%). Furthermore, wet cupping alone or combined with moxibustion effectively lowered the levels of inflammatory factors compared to medication: TNF-*α* (*n* = 120, MD = −6.99, 95% CI [−8.13, −5.85], *p* < 0.00001, I^2^ = 0%), IL-1β (*n* = 120, MD = −5.28, 95% CI [−6.91, −3.65], *p* < 0.00001, I^2^ = 48%), and IL-6 (*n* = 180, MD = −8.61, 95% CI [−13.24, −3.99], *p* = 0.0003, I^2^ = 81%).

**Conclusion:**

The efficacy of wet cupping therapy is comparable to that of high-potency steroids. Its combined use with medication or moxibustion appears to enhance effectiveness, reduce recurrence rates, and improve safety. However, due to the overall low grade of evidence for the identified outcomes and poor methodological quality, caution is advised when interpreting and applying these findings in clinical practice.

**Systematic review registration:**

https://www.crd.york.ac.uk/prospero, identifier: CRD42024524398.

## 1 Introduction

Neurodermatitis, a chronic and recurrent skin condition, stems from dysfunctions in the excitatory and inhibitory processes of the cerebral cortex (1). The itch–scratch cycle is a crucial neurophysiological mechanism of its pathogenesis; generally speaking, neurological disorders located in the cerebral cortex can induce endocrine disorders, leading to the release of catecholamines, acetylcholine, histamine, etc., causing itching (2). Chronic itching often triggers the patient’s urge to scratch, which in turn leads to neuronal inflammation, exacerbating and prolonging the itching (3, 4), further prompting the patients to reflexively scratch or rub their skin, exacerbating disruptions in the skin barrier, and leading to excessive corneum thickening, dry skin scaling, and chapping, ultimately resulting in skin lichenoid changes (5), and even to infection or malignant transformation in rare cases (6). With over 10% of the global population affected by neurodermatitis, onset typically occurs between the ages of 30 and 50, with a higher prevalence among women (7, 8). Topical corticosteroids are frequently efficacious as initial treatments for inflammatory dermatoses (6). They exhibit anti-proliferative, immunosuppressive, and hormonal activities. However, prolonged use increases the risk of skin atrophy and secondary infections (9), and a higher number of cases of hypersensitivity reactions have been reported in recent years due to their accessibility as over-the-counter (OTC) medications (10, 11). Another study suggested a potential association between topical corticosteroids and the risk of developing diabetes, which increased with cumulative dose and cumulative duration of use (12). Antihistamines are recommended as the first-line systemic treatment for neurodermatitis in China for anti-inflammatory and pruritus relief (13). Common side effects of first-generation and some second-generation antihistamines are known, such as unwanted sedative effects (fatigue, drowsiness, etc.) and anticholinergic effects (14, 15). They should also be used with caution in people with abnormal liver function. A large cohort study (16) reported that patients with hepatitis B and hepatitis C who took them were at an increased risk of developing liver cancer. For the above reasons, it is indispensable to seek other treatments to meet the management and safety of neurodermatitis.

Cupping, a procedure for physical stimulation of the skin, has been practiced for thousands of years, especially in East Asian and Islamic cultures (17, 18). In traditional Chinese medicine, practitioners make use of the power of flaming heating to create suction inside cups, enabling them to adhere quickly to acupuncture points or specific areas of the body (19). In contrast to dry cupping, wet cupping therapists use a sterile needle or scalpel to make a small incision in the superficial part of the skin and then place a cup over the incision and suck out the air inside, drawing out a small amount of blood or extracellular fluid (20). In recent years, interest in wet cupping has surged. It has not only been recognized as potentially beneficial for dermatological conditions (7, 21, 22), but Chinese scholars have also found that wet cupping is more advantageous than dry cupping in improving lower back pain (23). Despite numerous clinical trials conducted in recent decades to verify the efficacy of wet cupping, there is a lack of meta-analyses focusing on its effectiveness in treating neurodermatitis. Therefore, we aimed to evaluate the efficacy and safety of wet cupping for neurodermatitis through a meta-analysis of randomized controlled trials (RCTs), providing evidence-based support for its management.

## 2 Methods and analysis

This systematic review was conducted and reported in accordance with the PRISMA 2020 statement, an updated guideline for the reporting of systematic reviews (24), and the recommendations by the Cochrane Collaboration, an updated guideline for trusted systematic reviews—a new edition of the Cochrane Handbook for Systematic Reviews of Interventions (25). The systematic review was registered on the PROSPERO website^1^ with the registration number: CRD42024524398. The methods were not changed during the review unless otherwise denoted below.

### 2.1 Inclusion and exclusion criteria

#### 2.1.1 Types of studies

This study included only parallel group-designed RCTs published in English or Chinese. Non-RCTs, such as pre-and post-controlled studies, historically controlled studies, cohort studies, and cross-sectional studies, were excluded. The duplication of studies published elsewhere was also excluded.

#### 2.1.2 Types of participants

The study included adult patients (18 years or above) who met the diagnostic criteria for neurodermatitis issued by the Chinese Medical Association (13) or the European Dermatology Forum (26) or other authoritative academic organizations, regardless of sex, age, or ethnicity.

#### 2.1.3 Types of interventions

Wet cupping therapy. There are no restrictions on the materials of the cups, such as glass, plastic, and bamboo cans. The following needles were considered for bloodletting: plum-blossom needle, fire needle, three-edged needle, skin needle, and other types of acupuncture, regardless of the choice of acupoints or location. Adding any medication to the cups was not allowed. The study involved either wet cupping alone as intervention or wet cupping combined with other treatments in the control group.

#### 2.1.4 Types of outcome measures

##### 2.1.4.1 Primary outcomes

Total effective rate (TER), measured according to two common standards (27, 28), as cure rate + marked effective rate + remission rate. This was judged by calculating the clinical symptom score (including items such as itching, papules, erythema, and pigmentation).

##### 2.1.4.2 Secondary outcomes

Recurrence rate, incidence of adverse events, and levels of inflammatory factors (TNF-*α*, IL-1β, and IL-6).

### 2.2 Information sources and search strategies

Two reviewers independently performed a comprehensive search of the following electronic databases: four English databases [PubMed, Embase, Cochrane Central Register of Controlled Trials database (CENTRAL), and Web of Science (WOS)] and four Chinese databases (China National Knowledge Infrastructure (CNKI), SinoMed Database (CBM), VIP Database, and WF Database). In addition, three clinical trial registration platforms were searched: the World Health Organization (WHO) International Clinical Trials Registry Platform,^2^ the Chinese Clinical Trial Registry,^3^ and ClinicalTrials.gov^4^ on wet cupping for neurodermatitis from their inception to March 2024. The following key search terms were imposed: (a) clinical conditions: neurodermatitis, lichen simplex chronicus, etc.; (b) wet cupping therapy-related words: cupping therapy, wet cupping, cupping, bloodletting, bleeding cupping, etc.; and (c) trial type: an RCT. The terms “and” and “or” were combined between the search terms. The search strategies for these sources are shown in the Supplementary Material.

### 2.3 Selection process and data collection

**Figure 1:**
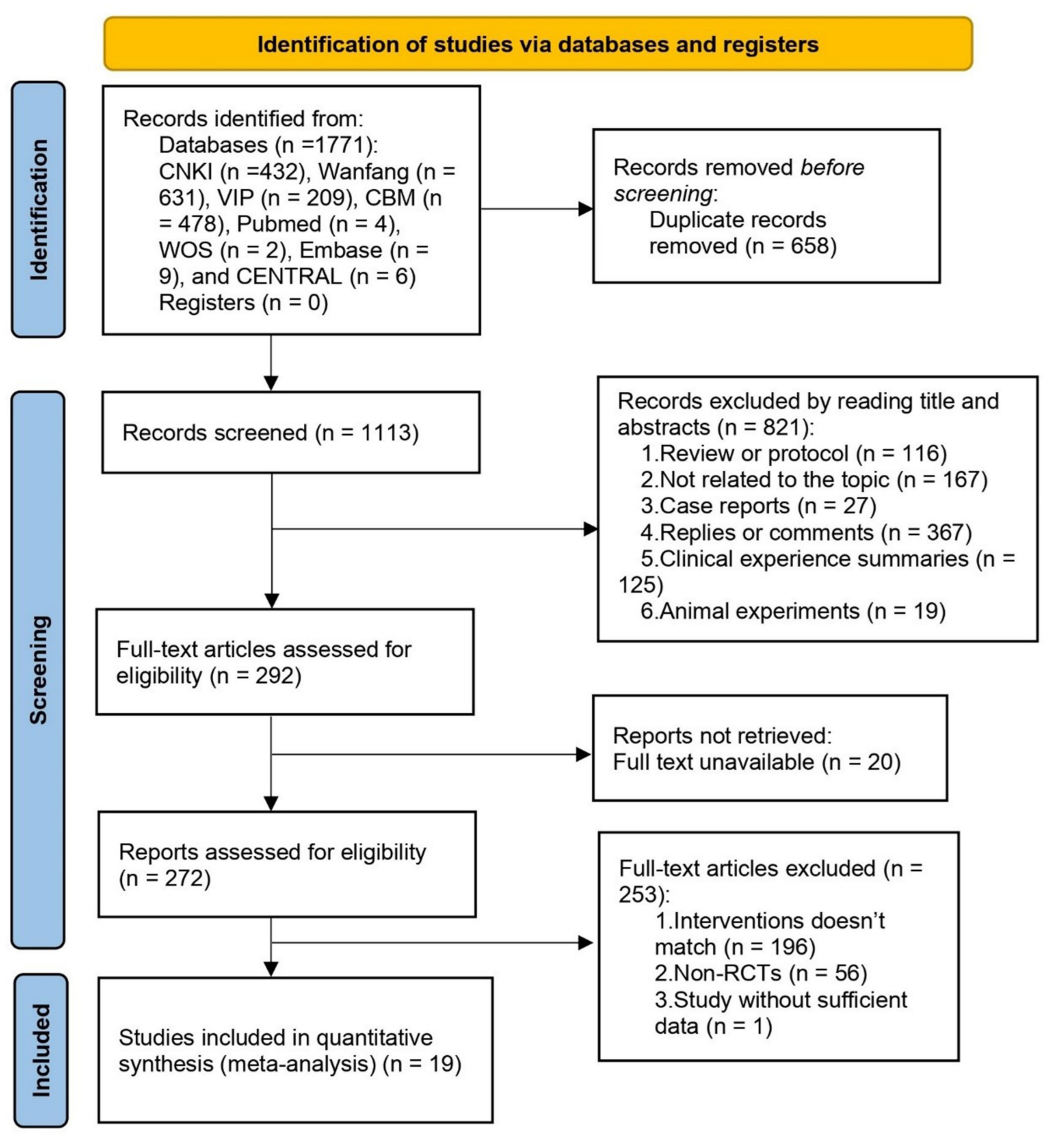
PRISMA flow diagram of the study selection process.

All investigators received professional, evidence-based medicine training to implement this systematic review. After excluding duplicate articles and uploading potentially eligible studies into Endnote V.20 software, two reviewers (LD and JC) independently screened the titles, abstracts, and keywords of all search items. They identified trials that met the above-mentioned inclusion criteria. Divergences between the two reviewers were resolved through discussion between the two. A third party also assisted in making the final decision. A PRISMA flowchart illustrating the study selection process is included in Figure 1.

Two reviewers (YZ and LD) independently performed the data collection using a standardized tabulation. The extracted elements were as follows: basic information (title, year of publication, first author, language, and country of implementation), participant characteristics at baseline (sample size, age, gender, and course of disease), study design (randomization method, allocation concealment, blinding procedures, diagnostic criteria, intervention frequency and duration, and acupoint composition), and outcomes. In the case of multi-arm RCTs, they were reclassified as dual-arm RCTs to ensure compatibility for result synthesis. The two reviewers conducted data extraction and cross-checking, and any discrepancies were resolved through consultation with the corresponding author.

### 2.4 Risk of bias assessment

Two independent reviewers (Z-FJ and XT) evaluated the quality of the included studies using the Cochrane Collaboration’s Risk of Bias Assessment tool (25). We evaluated the five items (bias in the randomization process, bias in deviations from the intended intervention, bias in missing outcome data, bias in the measurement of an outcome, and selection bias in the reported results). Each item was assessed and classified as high risk, low risk, or risk of some concern. The corresponding author were asked to resolve any divergences.

### 2.5 Data synthesis and assessment of heterogeneity

The statistical software ReviewManager V.5.4 was used for data analysis. The mean difference (MD) was used as the effect size for continuous variables, while the risk ratio (RR) was used for dichotomous variables. The 95% confidence interval (CI) was calculated. A significance level of *p*-value <0.05 was considered statistically significant. Statistical heterogeneity was assessed using the chi-squared tests and the Higgins I^2^ test. If *p*-value ≥0.1 and I^2^ ≤ 50%, indicating a high degree of homogeneity, a fixed-effects model was used for the pooled analysis. Conversely, if *p*-value <0.1 or I^2^ > 50%, indicating a significant degree of heterogeneity, a random-effects model was used. If there was significant heterogeneity between studies and the data were available, subgroups of the different cupping therapies and intervention forms were constructed to explore the potential causes of heterogeneity.

### 2.6 Reporting bias assessment

If more than 10 studies were included in the analysis, the funnel plot method and Egger’s test were used to evaluate publication biases. A *p*-value of <0.05 was considered indicative of significant publication bias. The analysis for publication bias was performed using STATA V.17.0.

### 2.7 Confidence in cumulative evidence

The Grading of Recommendations Assessment, Development, and Evaluation System (GRADEpro) evaluation tool^5^ was independently used by two reviewers (YZ and Z-FJ) to assess the quality of evidence in terms of five dimensions: risk of bias, inconsistency, indirectness, imprecision, and publication bias. The quality of evidence was classified as high, moderate, low, or very low. In the event of any disagreement, a third reviewer was consulted to facilitate consensus.

## 3 Results

### 3.1 Study selection

A total of 1,771 studies were initially identified by searching the databases using the keywords mentioned earlier. After stepwise screening, 19 RCTs with 1,505 participants were included in our meta-analysis (29–47). The flowchart of PRISMA is depicted in Figure 1.

### 3.2 Characteristics of the included trials

The main characteristics of the included RCTs are shown in Table 1. All studies were conducted in China between 1998 and 2023. Only one study was published in English (34), and the remaining studies were published in Chinese. All included studies were single-center, two-arm RCTs, of which 3 were master’s degree theses (37, 38, 41) and 16 were journal articles. The average age of the 1,505 participants ranged from 29 to 53 years and did not involve any minors or elderly patients. The course of the disease varied from 1 week to 20 years.

**Table 1:** Basic characteristics of the included studies.

Study	Country	Sample size (T/C)	Sex (male: female)		Age		Course of the disease (T/C)	Interventions		Dose or frequency	Follow-up time	Outcomes	Adverse events (T/C)	Needle
			T	C	T	C		T	C					
Ren et al. (47)	China	30/30	16:14	18:12	41.38 ± 3.51	41.52 ± 3.48	(6.42 ± 1.49) Y/(6.38 ± 1.52) Y	Wet cupping+moxibustion	Halometasone	Wet cupping+ moxibustion twice a week (3 weeks)	3 M and 6 M	TER, inflammatory factor levels, recurrence rate, and adverse events	Telangiectasia:1/telangiectasia:3;skin atrophy:2;precipitation of pigment:3	Plum-blossom needle
Wang et al. (29)	China	30/30	13:17	11:19	39.30 ± 12.11	41.96 ± 10.32	(20.23 ± 41.95) M/(23.73 ± 42.61) M	Wet cupping+moxibustion	Halometasone	Wet cupping+ moxibustion twice a week (3 weeks)	3 M and 6 M	TER, inflammatory factor levels, and recurrence rate	NR	Plum-blossom needle
Li et al. (44)	China	40/40	18:22	19:21	34.90 ± 8.27	35.21 ± 8.54	(12.33 ± 9.82) M/(13.21 ± 10.28) M	Wet cupping+clobetasol propionate	Clobetasol Propionate	Wet cupping +clobetasol propionate twice a week (2 weeks)	1 M	TER and recurrence rate	None	NR
Yu (37)	China	30/30	16:14	13:17	42.62 ± 9.20	41.57 ± 8.21	(12.86 ± 2.82) M/(13.26 ± 3.34) M	Wet cupping+acupuncture	Beclometasone dipropionate and camphor	Acupuncture+ wet cupping once every 6 days (12 days)	NR	TER	Hematoma and pain:2/none	Three-edged needle
Wang et al. (39)	China	60/60	NR	NR	46.20 ± 4.50	44.10 ± 3.60	(4.4 ± 2.2) Y/(4.1 ± 1.5) Y	Wet cupping+moxibustion	Triamcinolone	Wet cupping+ moxibustion every other day (4 weeks)	NR	TER and adverse events	Local stimulus response:1, hypopigmentation:2/local stimulus response:5, dermatrophy:2, and hypopigmentation:4	Plum-blossom needle
Zhang (38)	China	30/30	13:17	16:14	35.90 ± 9.67	36.67 ± 10.11	(33.93 ± 19.51) M/(30.97 ± 19.24) M	Wet cupping	Pevisone	Wet cupping once a week (8 weeks)	NR	TER, inflammatory factor levels, and adverse events	alcohol allergy:1/none	Three-edged needle
Feng (31)	China	30/30	18:12	17:13	41.70 ± 6.30	40.80 ± 6.10	NR	Wet cupping+ moxibustion	Triamcinolone	Wet cupping+moxibustion every other day (8 days)	NR	TER	NR	Plum-blossom needle
Yang and Wang (36)	China	43/43	21:22	23:20	30–51	32–52	(5.40 ± 3.60) Y/(4.90 ± 3.40) Y	Wet cupping+bloodletting therapy	Pevisone	Wet cupping+ bloodletting therapy every 3 days (60 days)	1Y	TER and recurrence rate	NR	Plum-blossom needle
Lin and Chen (43)	China	75/75	NR	NR	35.61 ± 1.73	33.97 ± 2.13	(3.12–14.15) Y/ (3.26–15.32) Y	Wet cupping	Halometasone	Wet cupping twice a week (30 days)	NR	TER	None	Plum-blossom needle
Guo (32)	China	30/30	12:18	13:17	41.9 ± 4.50	42.1 ± 4.50	NR	Wet cupping+moxibustion	Triamcinolone	Wet cupping+ moxibustion every other day (4 weeks)	NR	TER	NR	Plum-blossom needle
Chen et al. (30)	China	36/36	20:16	19:17	42.13 ± 2.13	41.00 ± 2.31	(7.86 ± 2.40) Y/(8.96 ± 2.39) Y	Wet cupping+halometasone	Halometasone	Wet cupping+ halometasone every 7 days (2 weeks)	NR	TER and adverse events	none	Disposable skin test needle
Zhang et al. (33)	China	25/23	15:10	12:11	52.36 ± 10.12	53.27 ± 10.14	(6.54 ± 3.51) Y/(5.87 ± 2.95)	Wet cupping+acupoint catgut embedding	Dexamethasone	Wet cupping+ acupoint catgut embedding every 7 days (4 weeks)	6 M	TER and recurrence rate	NR	Disposable injection needle
Hu et al. (35)	China	24/24	NR	NR	NR	NR	(2.1–12.6) Y/ (1.8–12.1) Y	Wet cupping	Halometasone	Frequency: unclear; period: 30 days	NR	TER	NR	Fire needle
Shao (34)	China	47/47	20:27	21:26	34.1 ± 5.0	33.8 ± 5.4	(61.3 ± 5.9 M/63.8 ± 5.9) M	Wet cupping+moxibustion	Triamcinolone	Wet cupping+ moxibustion once every 4 days (20 days)	NR	TER	NR	Plum-blossom needle
Zhang and Shi (45)	China	68/68	57:11	56:12	29.3 ± 8.5	30.2 ± 9.3	(1.8 ± 0.62) Y/(1.9 ± 0.66) Y	Wet cupping	Antihistamines	Wet cupping once every 3 days (30 days)	1 Y	TER and recurrence rate	NR	Plum-blossom needle
Zhang et al. (40)	China	23/23	13:10	12:11	49.91 ± 11.76	42.65 ± 13.28	(0.02–10) Y/(0.04–20) Y	Wet cupping+acupuncture	Traditional Chinese medicine decoction	Wet cupping+ acupuncture every other day (30 days)	NR	TER	NR	Plum-blossom needle
Li (42)	China	35/36	18:17	20:16	36.94 ± 11.29	35.06 ± 12.45	(14.67 ± 8.77) M/(13.32 ± 8.67) M	wet cupping	Mometasone	Wet cupping every other day (30 days)	1 M, 3 M, and 6 M	TER	Chromatosis:2/dermatrophy:3	Plum-blossom needle
Li and Yang (42)	China	48/32	28:20	10:22	36.1 ± 10.1	35.9 ± 10.5	(20.8 ± 11.3) M/(21.7 ± 11.5) M	Wet cupping+moxibustion	Triamcinolone	Wet cupping+ moxibustion every other day (4 weeks)	NR	TER	NR	Plum-blossom needle
Huang et al. (46)	China	60/60	39:21	40:20	Unclear	Unclear	Unclear	Wet cupping+bufexamac	Bufexamac	Wet cupping+ bufexamac every other day (12 days)	NR	TER	NR	Plum-blossom needle

T: test group; C: control group; M: month; Y: year; NR: not reported; TER: total effective rate.

### 3.3 Risk of bias

**Figure 2:**
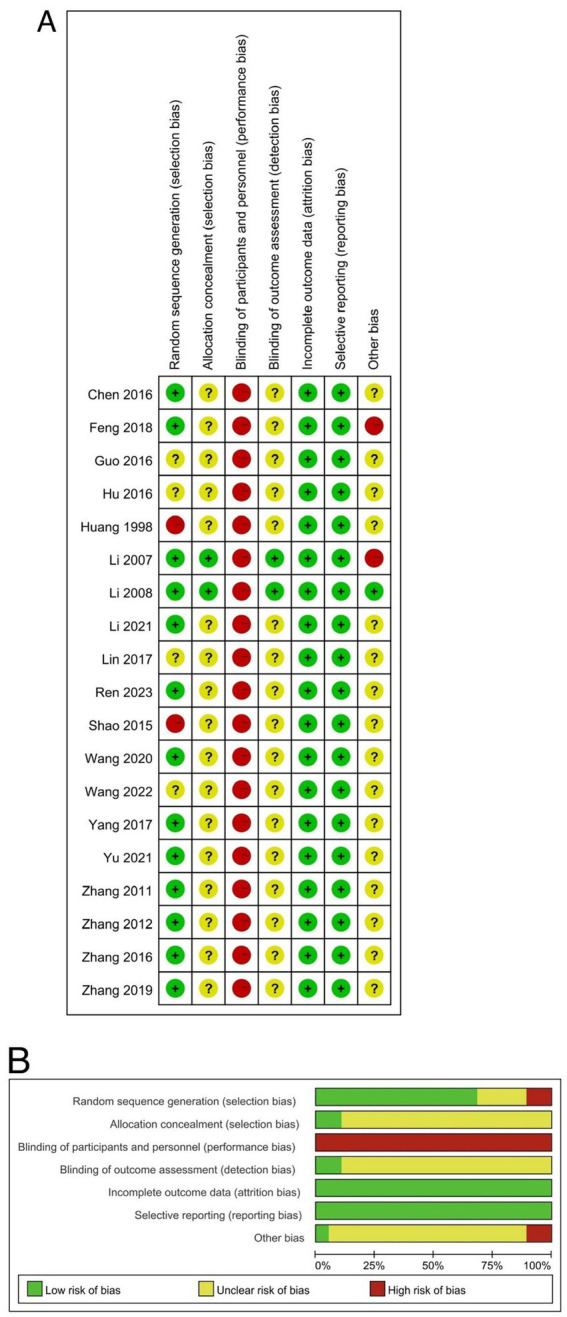
Risk of bias. **(A)** Bias of risk summary; **(B)** bias of risk graph.

The risk of bias assessment was conducted for each study, and the results are shown in Figure 2. All the included studies mentioned the term “randomized” or “random” in their methodology; 11 studies used random number tables, 1 study used statistical software to generate random numbers, and 1 study used the method of drawing lots for random allocation, while the other 6 studies did not mention specific randomization methods. Except for two studies (41, 42), none of the other 17 studies explicitly discussed the details of allocation concealment. Due to the nature of cupping therapy, it is unfeasible to blind the operators, so all studies received a “high risk” rating in the third item. Except for two studies (41, 42), the other 17 studies did not assess blinding in their outcomes, leading to “unclear” ratings. The outcome data of all the included studies were complete, and no studies were selectively reported. In terms of other biases, one study neither reported the total time patients received treatment nor assessed the time to treatment outcome (31), and another study had unbalanced baseline data (42), resulting in a high risk of bias.

### 3.4 Meta-analysis

#### 3.4.1 Total effective rate

##### 3.4.1.1 Wet cupping alone compared to medication alone

Five studies compared wet cupping alone with medication as an intervention. The pooled data analysis revealed no significant difference in the total effective rate between wet cupping and medication (*n* = 465, RR = 1.21, 95% CI [1.00, 1.47], *p* = 0.05, I^2^ = 83%).

**Figure 3:**
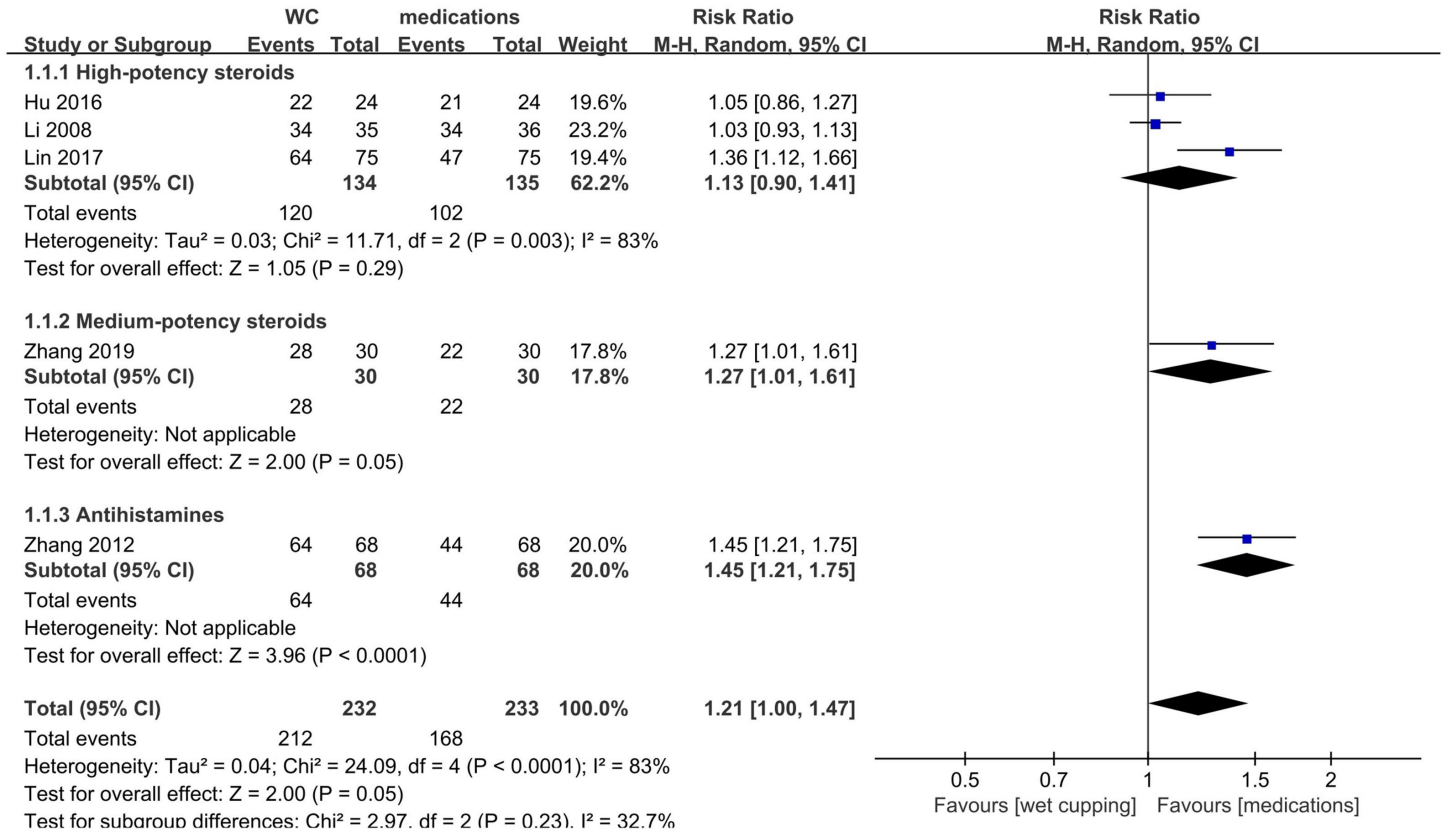
Forest plot of TER. Forest plot showing the effects of wet cupping (WC) compared to medication on the total effective rate in the treatment of neurodermatitis.

A subgroup analysis was performed based on different control medications; three studies (35, 41, 43) used high-potency steroids as controls, one study (38) used medium-potency steroids, and one (45) used antihistamines as controls (Figure 3). The results indicated that wet cupping was not more effective than high-potency steroids (*n* = 269, RR = 1.13, 95% CI [0.90, 1.41], *p* = 0.29, I^2^ = 83%), and the wet cupping compared to medium-potency steroids study reported no apparent benefit (*n* = 60, RR = 1.27, 95% CI [1.01, 1.61], *p* = 0.05). The other study comparing wet cupping with antihistamines showed statistically significant differences (*n* = 136, RR = 1.45, 95% CI [1.21, 1.75], *p* < 0.0001). Furthermore, we searched for sources of high heterogeneity in the high-potency steroid subgroup through sensitivity analysis and found that heterogeneity was significantly reduced after excluding the Lin and Chen (43) studies (*n* = 119, RR = 1.03, 95% CI [0.95, 1.13], *p* = 0.47, I^2^ = 0%).

##### 3.4.1.2 Wet cupping plus medication compared to medication alone

**Figure 4:**
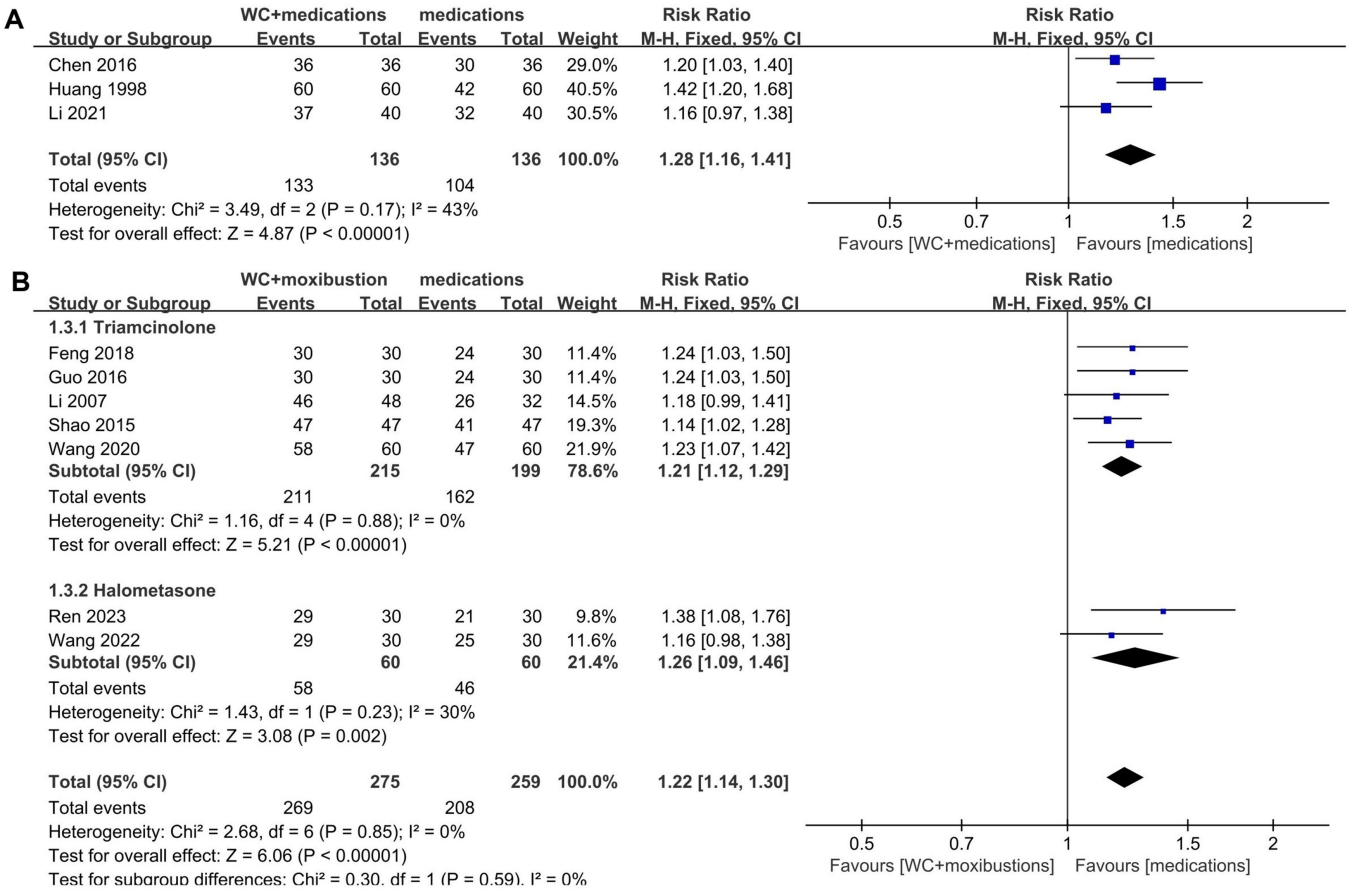
Forest plot of TER. **(A)** Forest plot showing the effects of wet cupping combined with medication compared to medication alone on the total effective rate in the treatment of neurodermatitis. **(B)** Forest plot showing the effects of wet cupping combined with moxibustion compared to medication alone on the total effective rate in the treatment of neurodermatitis.

Three studies had sufficient data to be pooled for meta-analysis; of these, two studies (30, 44) used corticosteroids in the control group, while one (46) used non-steroidal anti-inflammatory drugs. The pooled data analysis indicated that wet cupping and medication were associated with a higher total effective rate than medication alone (*n* = 272, RR = 1.28, 95% CI [1.16, 1.41], *p* < 0.00001, I^2^ = 43%; Figure 4A).

##### 3.4.1.3 Wet cupping plus moxibustion compared to medication

Seven studies reported their total effective rate for the combination of wet cupping with moxibustion compared to medication alone. Two of these studies (29, 47) used high-potency steroids (halometasone), while the other five studies (31, 32, 34, 39, 42) used medium-potency steroids (triamcinolone acetonide). The pooled analysis demonstrated that wet cupping therapy combined with moxibustion was more effective than medication alone (*n* = 534, RR = 1.22, 95% CI [1.14, 1.30], *p* < 0.00001, I^2^ = 0%; Figure 4B).

In the subgroup analysis, wet cupping combined with moxibustion was more effective than triamcinolone (*n* = 414, RR = 1.21, 95% CI [1.12, 1.29], *p* < 0.00001, I^2^ = 0%). Similarly, halometasone was not as effective as wet cupping combined with moxibustion (*n* = 120, RR = 1.26, 95% CI [1.09, 1.46], *p* = 0.002, I^2^ = 30%).

#### 3.4.2 Recurrence rate

**Figure 5:**
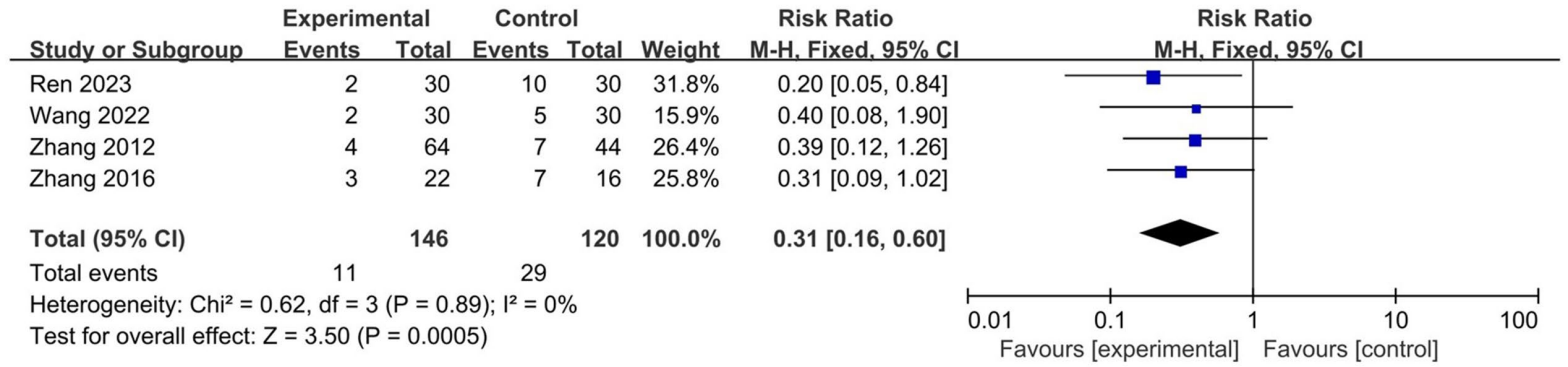
Forest plot of the recurrence rate at 6-month follow-up.

A total of four studies reported the recurrence rate at a 6-month follow-up (29, 33, 45, 47). The results of the meta-analysis showed that wet cupping alone or in combination with other treatments had a lower recurrence rate than other treatments (*n* = 266, RR = 0.31, 95% CI [0.16, 0.60], *p* = 0.0005, I^2^ = 0%; Figure 5).

#### 3.4.3 Adverse event incidence

**Figure 6:**
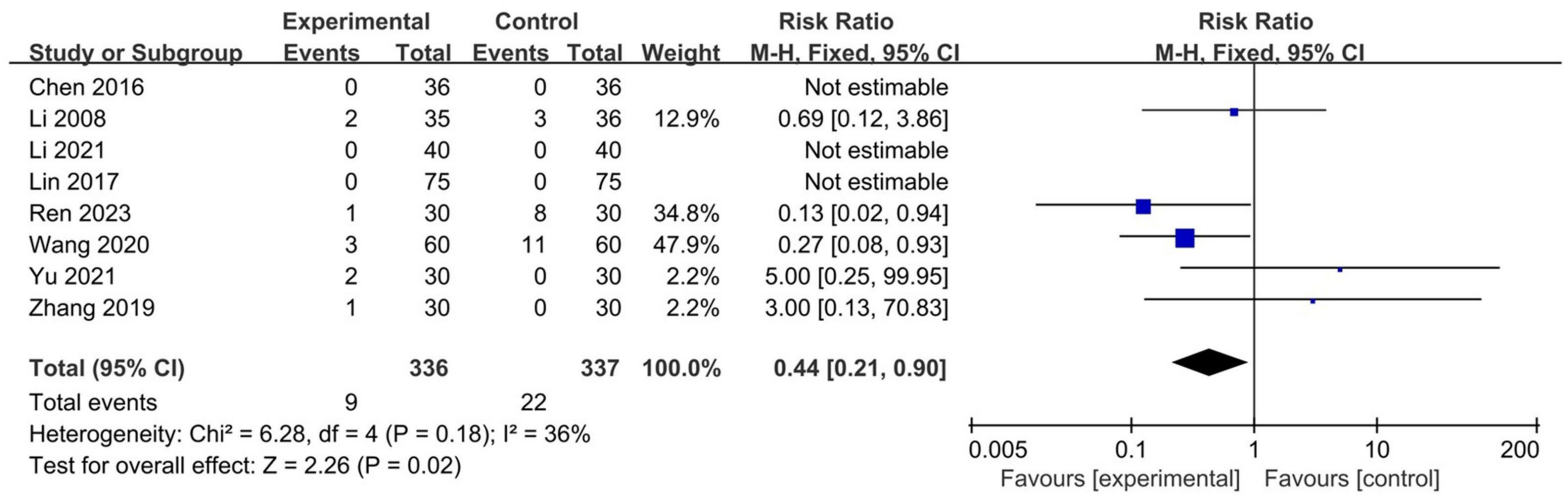
Forest plot of the incidence of adverse events.

A total of eight studies recorded adverse events, with three studies (30, 43, 44) reporting no adverse events in either the test or control groups. In five studies (37–39, 41, 47), the most common adverse events in the test group included hypopigmentation, subcutaneous hematoma, and pain. The main adverse events in the control group were skin atrophy, pigmentation, and local irritation. No serious adverse events were reported in either group. Interestingly, the results of the meta-analysis showed that wet cupping alone or in combination with other treatments was safer than medication (*n* = 673, RR = 0.44, 95% CI [0.21, 0.90], *p* = 0.02, I^2^ = 36%; Figure 6).

#### 3.4.4 Inflammatory factor levels

**Figure 7:**
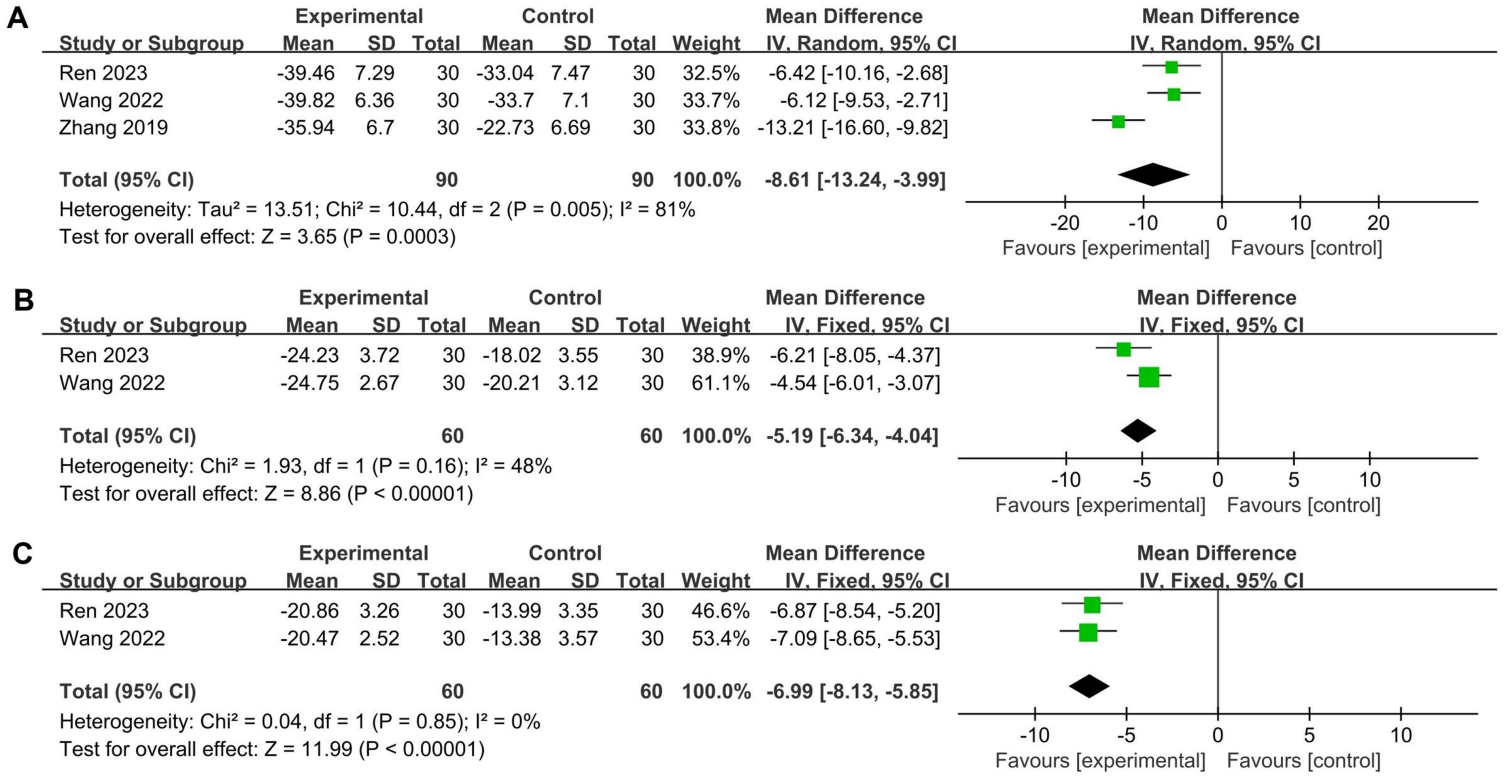
Forest plot of levels of inflammatory factors. **(A)** interleukin IL-6; **(B)** interleukin IL-1β; **(C)** tumor necrosis factor TNF-*α*.

Three studies reported the levels of inflammatory factors as an outcome (29, 38, 47). All of these studies reported decreases in the serum levels of interleukin IL-6, and two of the studies also reported decreases in the serum levels of interleukin IL-1β and tumor necrosis factor TNF-*α*. The meta-analysis of the pooled results indicated that compared to corticosteroids, wet cupping alone or combined with moxibustion had statistically significant differences in reducing the levels of inflammatory factors: TNF-α (*n* = 120, MD = −6.99, 95% CI [−8.13, −5.85], *p* < 0.00001, I^2^ = 0%), IL-1β (*n* = 120, MD = −5.28, 95% CI [−6.91, −3.65], *p* < 0.00001, I^2^ = 48%), and IL-6 (*n* = 180, MD = −8.61, 95% CI [−13.24, −3.99], *p* = 0.0003, I^2^ = 81%) (Figure 7).

Given the high heterogeneity of the statistical results for IL-6, a sensitivity analysis revealed a significant reduction after excluding the study by Zhang (38) (*n* = 120, MD = −6.26, 95% CI [−8.78, −3.74], *p* < 0.00001, I^2^ = 0%).

### 3.5 Publication bias

**Figure 8:**
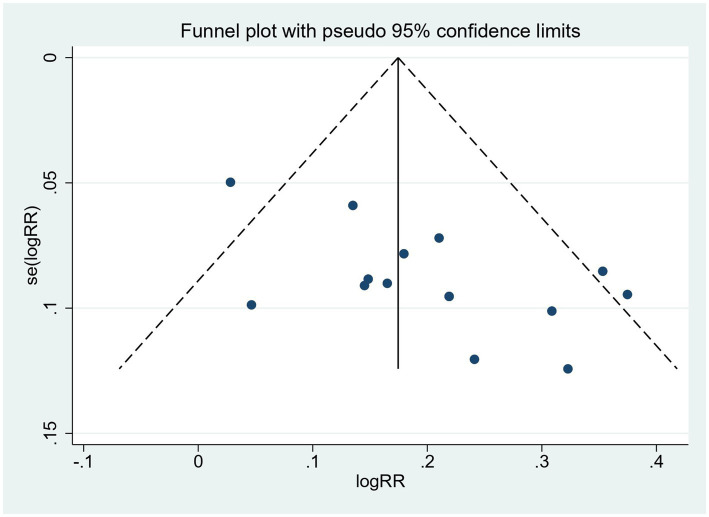
Funnel plots of included studies on total effective rate.

**Figure 9:**
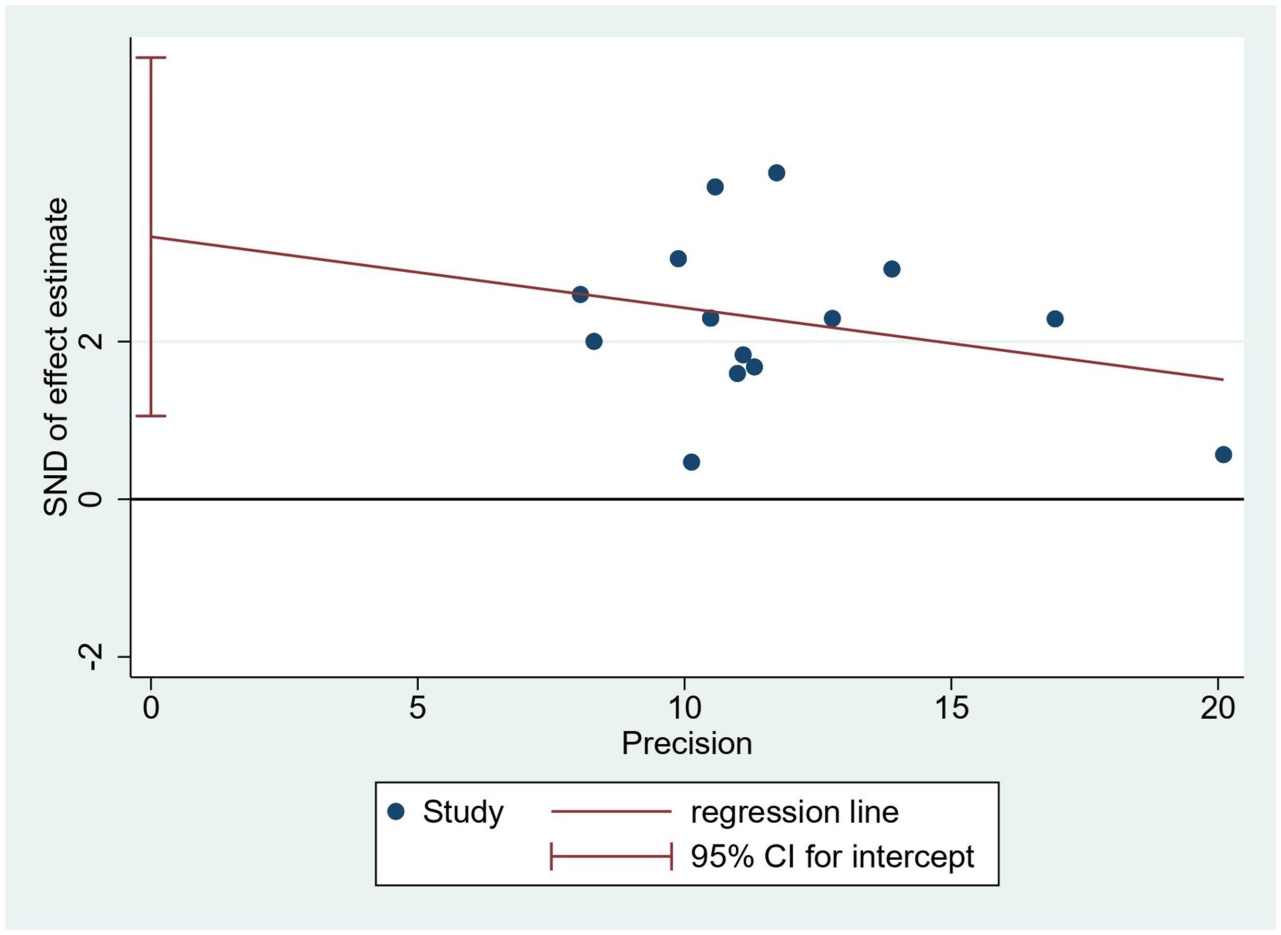
Egger’s test of the included studies on the total effective rate.

Since only the TER meets the requirement that the number of studies reporting outcomes be greater than 10, publication bias was tested only for the total effective rate. Visually, the funnel plot showed an asymmetric distribution of included studies, with four studies falling outside the pseudo 95% confidence interval (Figure 8). Furthermore, Egger’s test was conducted (Figure 9), and the results showed a total effective rate with *p* = 0.007, indicating the existence of significant publication bias. See Figures 8, 9 for details

### 3.6 Quality assessment of the evidence

Table 2 lists the quality assessment of each outcome of wet cupping and control in the meta-analysis. The GRADEpro system was used to assess the quality of evidence based on different intervention measures and outcomes. The results revealed six studies with low-quality evidence, two with very low-quality evidence, and none with high-quality or moderate-quality evidence. The majority of the studies had limitations in their experimental design, such as inadequate description of random sequence generation, allocation concealment, and blinding. Consequently, they were downgraded. In addition, the presence of publication bias in the majority of the studies contributed to their downgrading.

**Table 2:** GRADE evidence profile for studies in the meta-analysis.

Certainty assessment							Number of patients		Effect		Certainty	Importance
Number of studies	Study design	Risk of bias	Inconsistency	Indirectness	Imprecision	Publication bias	Experimental group	Control group	Relative (95% CI)	Absolute (95% CI)		
Wet cupping versus medication for TER												
5	RCT	Serious^a^	Serious^b^	Not serious	Not serious	Strongly suspected^c^	212/232 (91.4%)	168/233 (72.1%)	RR 1.21 (1.00 to 1.47)	151 more per 1,000 (from 0 fewer to 339 more)	⨁◯◯◯ Very low	Critical
Wet cupping + medication versus medication for TER												
3	RCT	Serious^a^	Not serious	Not serious	Not serious	Strongly suspected^c^	133/136 (97.8%)	104/136 (76.5%)	RR 1.28 (1.16 to 1.41)	214 more per 1,000 (from 122 more to 314 more)	⨁⨁◯◯ Low	Critical
Wet cupping + moxibustion versus medication for TER												
7	RCT	Serious^a^	Not serious	Not serious	Not serious	Strongly suspected^c^	269/275 (97.8%)	208/259 (80.3%)	RR 1.22 (1.14 to 1.30)	177 more per 1,000 (from 112 more to 241 more)	⨁⨁◯◯ Low	Critical
Wet cupping versus control for recurrence rate at 6-month follow-up												
6	RCT	Serious^a^	Not serious	Not serious	Not serious	Strongly suspected^d^	11/116 (7.5%)	29/120 (24.2%)	RR 0.31 (0.16 to 0.60)	167 fewer per 1,000 (from 203 fewer to 97 fewer)	⨁⨁◯◯ Low	Important
Wet cupping versus control for adverse events												
8	RCT	Serious^a^	Not serious	Not serious	Not serious	Strongly suspected^d^	9/336 (2.7%)	22/337 (6.5%)	RR 0.44 (0.21 to 0.90)	37 fewer per 1,000 (from 52 fewer to 7 fewer)	⨁⨁◯◯ Low	Important
Wet cupping versus control for inflammatory factor levels (TNF-α)												
2	RCT	Serious^a^	Not serious	Not serious	Not serious	Strongly suspected^d^	60	60	-	MD 6.99 lower (8.13 lower to 5.85 lower)	⨁⨁◯◯ Low	Important
Wet cupping versus control for inflammatory factor levels (IL-1β)												
2	RCT	Serious^a^	Not serious	Not serious	Not serious	Strongly suspected^d^	60	60	-	MD 5.28 lower (6.91 lower to 3.65 lower)	⨁⨁◯◯ Low	Important
Wet cupping versus control for inflammatory factor levels (IL-6)												
3	RCT	Serious^a^	Serious^b^	Not serious	Not serious	Strongly suspected^d^	90	90	-	MD 8.61 lower (13.24 lower to 3.99 lower)	⨁◯◯◯ Very low	Important

Question: wet cupping compared to controls for neurodermatitis. CI: confidence interval; RR: risk ratio; MD: mean difference.

a The design of the trial has a large bias in randomization, allocation concealment, or blinding.

b The credible interval overlaps less, the *p*-value of the heterogeneity test is small, and the I^2^ of the combined results is large.

c Funnel plot of the included studies for TER displays asymmetrical distribution, Egger’s test: *p* < 0.05.

d All studies from the same region/country.

## 4 Discussion

### 4.1 Main results

Neurodermatitis is one of the most prevalent and persistent pruritic dermatoses (8). Nocturnal paroxysmal pruritus, a hallmark of the condition, disrupts sleep and impairs daily functioning, imposing substantial psychological strain on patients (8, 48). Given the limitations of conventional treatments, there is an urgent need for complementary interventions. Our meta-analysis revealed that wet cupping alone did not show a higher TER than high-potency steroids, suggesting that both are equally effective. When used in combination with medication or moxibustion, wet cupping demonstrated superior efficacy compared to medication alone. Regarding secondary outcomes, wet cupping alone or in combination with other therapies can significantly reduce the recurrence rate and the incidence of adverse events.

We conducted an overall search of relevant literature databases and noted the rare use of dry cupping in neurodermatitis. Despite its potential benefits, such as regulating skin immunity and stimulating the release of inflammatory mediators (49), we hypothesized that the non-invasive nature of dry cupping may limit its effectiveness in fully stimulating the deeper layers of hypertrophic lichenified skin. The above reasons influenced our decision to exclude dry cupping from the systematic review. In contrast, wet cupping combines bloodletting and cupping as a physical stimulation procedure. This physical stimulation can cause local tissue congestion and decompression, accelerate blood circulation, and inflammatory exudate discharge (47); it also causes the release of histamine, stimulates phagocytosis, regulates the body’s immune network, and promotes the self-repair of damaged skin tissue (50). Among the studies in our systematic review, 12 used plum-blossom needles, 2 used three-edged needles, and 1 used fire needles, all of which are traditional Chinese acupuncture instruments. On the one hand, acupuncture may alleviate itching by exerting antihistamine effects, reducing itch mediator production, and modulating the brain network involved in the central transmission of itch (7, 51, 52). On the other hand, acupuncture can bolster the body’s stress response, induce denaturation of diseased tissue proteins, and activate macrophages and leukocytes to phagocytose necrotic tissue, thereby ameliorating skin lichenification (53, 54).

As an important effector of the immune system, the release and recruitment of inflammatory factors actively participate in and mediate cellular inflammatory responses (55). IL-6 has been reported to promote the activation and infiltration of inflammatory cells, exacerbating the inflammatory state of the skin and leading to keratinization and desquamation of the skin (56, 57). TNF-*α* is a tumor necrosis factor that induces the activation of macrophages and T cells, which, in turn, produce pro-inflammatory cytokines, etc. (58). The expression levels of IL-1β, IL-4, IL-6, and TNF-α in peripheral blood positively correlate with the severity of dermatitis (59) and may contribute to disease recurrence (60). Scholars have proposed that wet cupping reduces the aggregation of inflammatory factors by accelerating the metabolism of skin tissues and promoting the skin to repair the damaged tissues and the absorption of inflammatory substances to benefit neurodermatitis patients (50, 61). Our meta-analysis results indicated that, compared to the control group, wet cupping can significantly reduce the levels of inflammatory factors (TNF-α, IL-1β, and IL-6). However, at present, the specific mechanisms of wet cupping to reduce the serum inflammatory factor need to be further confirmed.

### 4.2 Limitations

Our review has important limitations that should be considered with caution. First, high heterogeneity appeared in the comparison of IL-6 and in the subgroup analysis of wet cupping alone versus high-potency steroids. The variations in study designs, intervention frequencies, outcome measurement intervals, skin lesion sites, or unaccounted confounding factors across comparisons may introduce significant clinical heterogeneity. This may lead to the unreliability of our pooled analysis results. Second, all the studies included in this meta-analysis were completed in China and multicenter trials were lacking, which may affect the quality of evidence in this analysis. Third, due to the nature of cupping therapy, blinding of cupping operators and participants was not feasible, which may have affected the reporting of treatment results in favor of wet cupping. Fourth, 4 studies did not provide information on random sequence generation, 2 used incorrect randomization methods, and 17 studies did not clarify whether allocation concealment was applied. In addition, significant publication bias was evident. Consequently, the overall quality of evidence in these studies may not be sufficient to extrapolate the findings to broader contexts. Fifth, the primary outcome used in the included studies was the total effective rate, which is not an internationally recognized standard. Furthermore, trial details were not comprehensively reported under standard reporting guidelines such as CONSORT. Sixth, the variability in total treatment time and frequency of cupping in each study is often determined by the cupping practitioner’s individual experience, resulting in varying efficacy and outcomes within the same intervention framework.

### 4.3 Implications for practice and research

Our data show that wet cupping is effective and that patients may gain additional benefits by adding wet cupping to corticosteroid or antihistamine therapy. This finding offers patients more treatment options and aids physicians make informed decisions. For patients who have long relied on medication to manage their condition and suffer from its side effects, wet cupping could be a viable alternative. Our research results suggest that wet cupping is relatively safe; however, it is important to adhere to operating specifications to prevent skin trauma or infection. We recommend the establishment of standardized guidelines for cupping therapy to facilitate its clinical promotion and application, despite being somewhat challenging.

The neuro-anxiety state plays a key role in the pathogenesis of neurodermatitis, with a demonstrated correlation between patients’ anxiety levels and the severity of pruritus (48). However, the included studies failed to incorporate relevant indicators to assess patients’ mental state and improvement. Future studies should consider incorporating the Hamilton Anxiety Scale (HAMA) and the Quality of Life Questionnaire (QOL) as outcomes to evaluate an individual’s mental status and overall quality of life. This would help define the impact of mental state on neurodermatitis patients so that appropriate interventions or research protocols can be prescribed.

Overall, the mechanism of action of wet cupping therapy on neurodermatitis remains unclear, necessitating further basic research to complement the clinical evidence. Given the limited quality of evidence in the studies reviewed, we anticipate more SRs or RCTs with reasonable designs and appropriate methods to validate our findings.

## 5 Conclusion

This review highlights the efficacy of wet cupping in the treatment of neurodermatitis in terms of total effective rate, recurrence, and safety. The originality of our study lies in comparing the efficacy of wet cupping when used alone, in combination with medication, or alongside moxibustion, against the efficacy of medication to determine its significance in the management of neurodermatitis.

Our findings revealed no difference in the efficacy of wet cupping compared to high-potency steroids. However, wet cupping may improve treatment efficacy when it is used as an adjunctive therapy to corticosteroids or alongside moxibustion, reduce recurrence rates and adverse event incidence, and significantly decrease inflammatory factor levels (TNF-*α*, IL-1β, and IL-6). Due to the poor methodological quality of allocation concealment and blinding implementation in these studies, along with the overall low grade of evidence, the results should be interpreted and applied in practice with caution.

## Data Availability

The original contributions presented in the study are included in the article/supplementary material, further inquiries can be directed to the corresponding author/s.

## Footnotes

^1^ https://www.crd.york.ac.uk/prospero/

^2^ http://www.who.int/ictrp/en/

^3^ http://www.chictr.org/en/

^4^ https://www.clinicaltrials.gov/

^5^ https://www.gradepro.org/
