# Supplementary material for: Efficacy and safety of wet cupping in the treatment of neurodermatitis: a systematic review and meta-analysis
Source: Front Med (Lausanne). 2024 Dec 19;11:1478073. doi: 10.3389/fmed.2024.1478073 (PMC11693718; doi:10.3389/fmed.2024.1478073)
Supplement: Supplementary file 1 [file Table_1.docx]

**Search checklist of each database**

**PubMed**

#1 "Neurodermatitis"[Mesh]

#2 " neurodermatitis"[Title/Abstract] OR "lichen simplex chronics"[Title/Abstract] OR "Neurodermatitis, Localized"[Title/Abstract] OR "Localized Neurodermatitis"[Title/Abstract] OR "Neurodermatitis, Circumscribed"[Title/Abstract] OR "Circumscribed Neurodermatitis"[Title/Abstract]

#3 #1 OR #2

#4 "Cupping therapy"[Mesh]

#5 "cupping therapy"[Title/Abstract] OR "wet cupping"[Title/Abstract] OR "cupping"[Title/Abstract] OR "blood-letting*"[Title/Abstract] OR "bleeding cupping*"[Title/Abstract] OR "pricking blood"[Title/Abstract] OR "fire needle"[Title/Abstract] OR "plum-blossom needle"[Title/Abstract] OR "three-edged needle"[Title/Abstract] OR "skin needle"[Title/Abstract]

#6 #4 OR #5

#7 "clinical trials, randomized"[Mesh Terms] OR "controlled clinical trials, randomized"[Mesh Terms] OR "clinical trials as topic"[MeSH Terms] OR "random allocation"[MeSH Terms] OR "therapeutic use"[MeSH Subheading]

#8 ("clinical"[Title/Abstract] AND "trial"[Title/Abstract]) OR "clinical trial"[Publication Type] OR "random*"[Title/Abstract]

#9 #7 OR #8

#10 #3 AND #6 AND #9

**EMBASE**

#1 'Neurodermatitis'/exp/mj

#2 'neurodermatitis':ti,ab,kw OR 'lichen simplex chronics':ti,ab,kw OR 'Neurodermatitis, Localized':ti,ab,kw OR 'Localized Neurodermatitis':ti,ab,kw OR 'Neurodermatitis, Circumscribed':ti,ab,kw OR 'Circumscribed Neurodermatitis':ti,ab,kw

#3 #1 OR #2

#4 'Cupping therapy'/exp/mj

#5 'cupping therapy':ti,ab,kw OR 'wet cupping':ti,ab,kw OR 'cupping':ti,ab,kw OR 'blood-letting':ti,ab,kw OR 'bleeding cupping':ti,ab,kw OR 'pricking blood':ti,ab,kw OR 'fire needle':ti,ab,kw OR 'plum-blossom needle':ti,ab,kw OR 'three-edged needle':ti,ab,kw OR 'skin needle':ti,ab,kw

#6 #4 OR #5

#7 'clinical trials, randomized'/exp/mj OR 'controlled clinical trials, randomized'/exp/mj OR 'clinical trials as topic'/exp/mj OR 'random allocation'/exp/mj

#8 clinical:ti,ab,kw OR trial:ti,ab,kw OR random*:ti,ab,kw

#9 #7 OR #8

#10 #3 AND #6 AND #9

**Web of science**

TS=(‘Neurodermatitis’ OR ‘lichen simplex chronics’ OR ‘Neurodermatitis, Localized’ OR ‘Localized Neurodermatitis’ OR ‘Neurodermatitis, Circumscribed’ OR ‘Circumscribed Neurodermatitis’) AND TS=(cupping therapy’ OR ‘wet cupping’ OR ‘cupping’ OR ‘blood-letting’ OR ‘bleeding cupping’ OR ‘pricking blood’ OR ‘fire needle’ OR ‘plum-blossom needle’ OR ‘three-edged needle’ OR ‘skin needle’) AND TS=(‘random*’ OR ‘clinical’ OR ‘trial’ )

**Cochrane Library**

#1 Mesh descriptor: [Neurodermatitis]explode all trees

#2 neurodermatitis:ti,ab,kw OR lichen simplex chronics:ti,ab,kw OR Neurodermatitis, Localized:ti,ab,kw OR Localized Neurodermatitis:ti,ab,kw OR Neurodermatitis, Circumscribed:ti,ab,kw OR Circumscribed Neurodermatitis:ti,ab,kw

#3 #1 OR #2

#4 Mesh descriptor: [Cupping therapy] explode all trees;

#5 cupping therapy:ti,ab,kw OR wet cupping:ti,ab,kw OR cupping:ti,ab,kw OR blood-letting:ti,ab,kw OR bleeding cupping:ti,ab,kw OR pricking blood:ti,ab,kw OR fire needle:ti,ab,kw OR plum-blossom needle:ti,ab,kw OR three-edged needle:ti,ab,kw OR skin needle:ti,ab,kw

#6 #4 OR #5

#7 Mesh descriptor: [clinical trials, randomized]

#8Mesh descriptor: [controlled clinical trials, randomized]

#9Mesh descriptor: [clinical trials as topic]

#10Mesh descriptor: [random allocation] explode all trees;

#11 clinical:ti,ab,kw OR trial:ti,ab,kw OR random*:ti,ab,kw

#12 #7 OR #8 OR #9 OR #10 OR #11

#13 #3 AND #6 AND #12

**CNKI**

(TKA=(‘拔罐’+‘刺络拔罐’+‘刺络’+‘拔罐放血’+‘放血’+‘刺血’+‘叩刺’+‘点刺’+‘梅花针’+‘皮肤针’+‘火针’+‘燔针’+‘三棱针’)) AND (TKA=(‘神经性皮炎’+‘牛皮癣’+‘慢性单纯性苔藓’+‘摄领疮’)) AND (TKA=(‘随机’+‘对照’) OR SU=(‘随机对照试验’))

**WF**

(题名或关键词:(神经性皮炎 OR 牛皮癣 OR 慢性单纯性苔藓 OR 摄领疮)) and (题名或关键词:(拔罐 OR 刺络拔罐 OR 刺络 OR 拔罐放血 OR 放血 OR 刺血 OR 叩刺 OR 点刺 OR 梅花针 OR 皮肤针 OR 火针 OR 燔针 OR 三棱针)) and (题名或关键词:(随机 OR 对照) OR 主题:(随机对照试验))

**Chongqing VIP**

M=(神经性皮炎 OR 牛皮癣 OR 慢性单纯性苔藓 OR 摄领疮) and M=(拔罐 OR 刺络拔罐 OR 刺络 OR 拔罐放血 OR 放血 OR 刺血 OR 叩刺 OR 点刺 OR 梅花针 OR 皮肤针 OR 火针 OR 燔针 OR 三棱针) and R=(随机 OR 对照)

**CBM**

1 "神经性皮炎" [加权:扩展]

2 "神经性皮炎"[常用字段:智能] OR "牛皮癣"[常用字段:智能] OR "慢性单纯性苔藓"[常用字段:智能] OR "摄领疮"[常用字段:智能]

3 1 OR 2

4 "拔罐"[加权:扩展]

5 "拔罐"[常用字段:智能] OR "刺络拔罐"[常用字段:智能] OR "刺络"[常用字段:智能] OR "拔罐放血"[常用字段:智能] OR "放血"[常用字段:智能] OR "刺血"[常用字段:智能] OR "叩刺"[常用字段:智能] OR "点刺"[常用字段:智能] OR "梅花针"[常用字段:智能] OR "皮肤针"[常用字段:智能] OR "火针"[常用字段:智能] OR "燔针"[常用字段:智能] OR "三棱针"[常用字段:智能]

6 4 OR 5

7 "随机对照试验"[不加权:扩展]

8 "随机"[常用字段:智能] OR "对照"[常用字段:智能]

9 7 OR 8

10 3 AND 6 AND 9
